# The role of the small intestine in the development of dietary fat-induced obesity and insulin resistance in C57BL/6J mice

**DOI:** 10.1186/1755-8794-1-14

**Published:** 2008-05-06

**Authors:** Nicole JW de Wit, Hanneke Bosch-Vermeulen, Philip J de Groot, Guido JEJ Hooiveld, Mechteld M Grootte Bromhaar, Jenny Jansen, Michael Müller, Roelof van der Meer

**Affiliations:** 1 Nutrition, Metabolism & Genomics Group, Division of Human Nutrition, Wageningen University, Wageningen, The Netherlands; 2 Nutrigenomics Consortium, TI Food & Nutrition, Wageningen, The Netherlands; 3 NIZO Food Research, Ede, The Netherlands

## Abstract

**Background:**

Obesity and insulin resistance are two major risk factors underlying the metabolic syndrome. The development of these metabolic disorders is frequently studied, but mainly in liver, skeletal muscle, and adipose tissue. To gain more insight in the role of the small intestine in development of obesity and insulin resistance, dietary fat-induced differential gene expression was determined along the longitudinal axis of small intestines of C57BL/6J mice.

**Methods:**

Male C57BL/6J mice were fed a low-fat or a high-fat diet that mimicked the fatty acid composition of a Western-style human diet. After 2, 4 and 8 weeks of diet intervention small intestines were isolated and divided in three equal parts. Differential gene expression was determined in mucosal scrapings using Mouse genome 430 2.0 arrays.

**Results:**

The high-fat diet significantly increased body weight and decreased oral glucose tolerance, indicating insulin resistance. Microarray analysis showed that dietary fat had the most pronounced effect on differential gene expression in the middle part of the small intestine. By overrepresentation analysis we found that the most modulated biological processes on a high-fat diet were related to lipid metabolism, cell cycle and inflammation. Our results further indicated that the nuclear receptors Ppars, Lxrs and Fxr play an important regulatory role in the response of the small intestine to the high-fat diet. Next to these more local dietary fat effects, a secretome analysis revealed differential gene expression of secreted proteins, such as Il18, Fgf15, Mif, Igfbp3 and Angptl4. Finally, we linked the fat-induced molecular changes in the small intestine to development of obesity and insulin resistance.

**Conclusion:**

During dietary fat-induced development of obesity and insulin resistance, we found substantial changes in gene expression in the small intestine, indicating modulations of biological processes, especially related to lipid metabolism. Moreover, we found differential expression of potential signaling molecules that can provoke systemic effects in peripheral organs by influencing their metabolic homeostasis. Many of these fat-modulated genes could be linked to obesity and/or insulin resistance. Together, our data provided various leads for a causal role of the small intestine in the etiology of obesity and/or insulin resistance.

## Background

Metabolic syndrome is a multi-component condition associated with a high risk of type 2 diabetes mellitus and cardiovascular disease [1]. In industrialized societies, approximately 20–40% of the population is affected by the metabolic syndrome and its incidence is expected to rise even further in the next decades [2]. Obesity and insulin resistance are two major risk factors underlying the metabolic syndrome. Obesity is considered the principal causal factor of insulin resistance, which is the pivotal metabolic disturbance in the metabolic syndrome [3].

Lifestyle factors, such as nutrition and limited physical activity, are known to contribute to the pathogenesis of obesity and insulin resistance. The association between development of these disorders and excessive intake of dietary fat is frequently studied, especially in obesity-prone C57BL/6J mice [4-8]. Most of these studies focused on the physiology and underlying molecular mechanisms in liver, skeletal muscle and adipose tissue, as these organs are target organs of insulin-modulated metabolism [9-11]. However, there is growing evidence that also the small intestine can play a role in the etiology of obesity and/or insulin resistance, as it serves as a gatekeeper at the physical interphase between body and diet. Efficient absorption and metabolic processing of nutrients is a major function of the small intestine, in which various nuclear receptors are likely to play an important regulatory role. Additionally, the enterocytes in the small intestine are responsible for sensing of luminal contents that are modulated by the diet. As a result of this sensing, the small intestine secretes signaling molecules, such as gut hormones and pro- and anti-inflammatory cytokines, to which liver, muscle and adipose tissue respond by modulating their metabolism to keep homeostatic control. Small intestinal factors that are previously reported to contribute to development of metabolic syndrome are gut hormones affecting satiety and glucose homeostasis [12,13], diminished fatty acid oxidative capacity of enterocytes [5] and gut microbiota-mediated modulations [6,14].

In this study, microarray analysis of small intestinal mucosa was performed to explore the role of the small intestine in development of dietary fat-induced obesity and/or insulin resistance in C57BL/6J mice. We determined high-fat diet-induced molecular changes along the longitudinal axis of the small intestine, at different time points during a Western-style humanized high-fat diet intervention. We analyzed the biological processes in the small intestine that are affected by feeding a high-fat diet and determined the involvement of nuclear receptors in gene expression changes. Beside the molecular changes that provoke a more local effect in the small intestine, we further studied the differential gene expression of (novel) small intestinal secreted proteins that are able to induce systemic effects. Therefore, an additional secretome analysis was performed. Finally, we linked the molecular changes found in the small intestine after high-fat diet feeding to development of obesity and insulin resistance.

## Methods

### Animals and diets

Male C57BL/6J mice (age 4 weeks) were purchased from Harlan (Horst, The Netherlands) and were housed in the light and temperature-controlled animal facility (12/12 (light/dark), 20°C) of Wageningen University. They had free access to water and received standard laboratory chow (RMH-B, Arie Blok BV, Woerden, The Netherlands) for two weeks, followed by the low-fat diet for three weeks to adapt to the purified diets. All experiments were approved by the Ethical Committee on animal testing of Wageningen University. To investigate the effect of dietary fat on development of obesity and insulin resistance and on small intestinal gene expression in C57BL/6J mice, we used low-fat (reference) and high-fat diets that are based on 'Research Diets' formulas D12450B/D12451, with adaptations regarding type of fat (palm oil in stead of lard) and carbohydrates to mimic the fatty acid and carbohydrate composition of the average human diet in Western societies (Research diet services, Wijk bij Duurstede, The Netherlands). Thus, the high-fat diet mimics the ratio of saturated to monounsaturated to polyunsaturated fatty acids (40:40:20) in a human diet. The complete composition of the diets is given in Additional file 1. It should be noted that in these diets the energy density of all nutrients, except fat and starch, is equal.

At the start of the diet intervention, the mice were divided into two groups and were fed a powdered high- or a low-fat purified diet. After 2, 4, and 8 weeks of diet intervention, 6 mice per diet group, per time point were sacrificed after they were anaesthetized with a mixture of isofluorane (1.5%), nitrous oxide (70%) and oxygen (30%). All mice were sacrificed in the postprandial state and diurnal variability was avoided by harvesting small intestines at the same time of the day for both diet groups. The small intestines were divided into three equal parts along the longitudinal axis (proximal, middle and distal part of the small intestine). Small intestinal epithelial cells were scraped, snap-frozen in liquid nitrogen, and stored at -80°C until RNA isolation. Body weight was recorded weekly. The mice that were sacrificed after 8 weeks of diet intervention were all subjected to an oral glucose tolerance test (OGTT) at week 7. Therefore, after 6-hours fasting, all mice received 0.5 ml of a 20% glucose solution via an oral gavage and blood glucose was measured after 15, 30, 45, 60, 90 and 150 minutes using Accu-Chek blood glucose meters (Roche Diagnostics, Almere, The Netherlands). To determine food intake, non-absorbable chromic oxide was supplemented to the diets for one week (week 5 of diet intervention). At the end of this week feces was quantitatively collected during 48 hours and fecal chromic oxide levels were determined as previously described [15]. These fecal chromic oxide levels were then used to calculate the energy intake per mouse per day on a high-fat and low-fat diet. An outline of this study design is presented in Additional file 2.

For immunohistochemical analysis, an identical low-fat and high-fat diet intervention study was performed for 2 weeks (n = 12 per diet). Small intestines were again excised, divided into three equal parts, cut open longitudinally, and washed with PBS. Thereafter, the small intestinal parts were fixed in 10% buffered formalin and embedded in paraffin.

### RNA isolation

Total RNA was isolated using TRIzol reagent (Invitrogen, Breda, The Netherlands) according to the manufacturer's instructions. The isolated RNA was further column-purified using the SV total RNA isolation system (Promega, Leiden, The Netherlands). RNA concentration was measured on a NanoDrop ND-1000 UV-Vis spectrophotometer (Isogen, Maarssen, The Netherlands) and analyzed on a bioanalyzer (Agilent Technologies, Amsterdam, the Netherlands) with 6000 Nano Chips according to the manufacturer's instructions.

### Microarray hybridization and analysis

Per time point, equal amounts of total RNA of six mice per diet group were pooled for the proximal, middle and distal part of the small intestine. These pooled RNA samples were hybridized to Mouse genome 430 2.0 arrays (Affymetrix, Santa Clara, CA, USA). Detailed methods for the labelling and subsequent hybridizations to the arrays are described in the eukaryotic section in the GeneChip Expression Analysis Technical Manual Rev. 3 from Affymetrix, which is available upon request. Arrays were scanned on an Affymetrix GeneChip Scanner 3000. For microarray data analysis Microarray Analysis Suite 5.0 (MAS 5.0) was used, which is also provided by Affymetrix. Comparison analysis by MAS 5.0 was performed to determine the dietary fat-induced differential gene expression for each part of the small intestine per time point. To indicate the magnitude and direction of differential gene expression, MAS 5.0 software provides signal log ratio's (SLR). If the SLR is equal to or greater than 0, fold change is obtained with +2^SLR^, otherwise with -2^-SLR^. Array data have been submitted to the Gene Expression Omnibus, accession number GSE8582.

To determine overrepresentation of Gene Ontology (GO) Biological Process subsets upon high-fat diet intervention, an ErmineJ overrepresentation analysis (ORA) was performed [16]. Gene score files that were used as input contained the 'change p-values' of all probes sets provided by MAS 5.0 comparison analysis. By setting the Gene score threshold to a p-value of 0.0025, only significantly differentially expressed genes were included in the ORA analyses. Moreover, only GO subsets that contained between 2 and 125 genes were taking into account. The ErmineJ software generally uses Benjamini-Hochberg correction of p-values to determine which gene sets are selected with a particular false discovery rate (FDR). The FDR is considered a rapid and reasonable guide to which gene sets are likely to be of highest interest [16].

Heat map diagrams visualizing SLR of differentially expressed genes were made using Spotfire DecisionSite software by applying hierarchical clustering.

### cDNA synthesis and real-time quantitative PCR

For each individual sample, single-stranded complementary DNA (cDNA) was synthesized from 1 μg of total RNA using the Reverse transcription system (Promega, Leiden, The Netherlands) following the supplier's protocol. cDNA was PCR amplified with Platinum Taq DNA polymerase (all reagents were from Invitrogen). Primer sequences (see Additional file 3) were retrieved from the online PrimerBank database [17], or otherwise designed using the Primer3 program [18]. Primers were tested for specificity by BLAST analysis. Real-time quantitative PCR (qPCR) was performed using SYBR green and a MyIQ thermal cycler (Bio-Rad laboratories BV, Veenendaal, The Netherlands). The following thermal cycling conditions were used: 2 min at 94°C, followed by 40 cycles of 94°C for 15 s and 60°C for 45 s. PCR reactions to validate dietary fat-induced differential gene expression were performed in duplicate and all samples were normalized to 18S and cyclophilin A expression. To analyse basal gene expression of nuclear receptors along the longitudinal axis of the small intestine we only normalized for cyclophilin A, as we found significant differences for 18S expression between the proximal, middle and distal parts of the small intestine.

### Immunohistochemistry

Four-micrometer sections of paraffin-embedded distal part of the small intestine were mounted on Superfrost microscope slides. These sections were dewaxed in xylene and rehydrated in a series of graded alcohols. To block endogenous peroxidase activity, slides were incubated with 3% H_2_O_2 _for 20 minutes. Antigen retrieval was performed by placing the slides in citrate buffer (pH 6.0) and heat them in a microwave oven 5 min 700 W (without lid) and 4 times 5 min 500 W (with lid). After cooling down to room temperature, the sections were briefly washed with PBS. Prior to staining, a 20 minutes preincubation was performed using 20% normal goat serum (Vector Laboratories, Burlingame, CA, USA). The sections were stained in a three-step procedure utilizing the following incubations: overnight incubation at 4°C with rat monoclonal antibodies against Ki67 (Clone TEC-3) (DakoCytomation B.V., Heverlee, Belgium), diluted 1:200 in PBS. Thereafter, the sections were incubated with a biotinylated goat-anti-rat for 30 minutes, followed by 45 minutes incubation with peroxidase-labelled avidin-biotin complex (Vector Laboratories). Between all incubations, sections were washed three times in PBS. Diaminobenzidine tetrahydrochloride (DAB, Vector Laboratories) was used as substrate to visualize the bound antibodies. After counterstaining with Meyer's hematoxylin, sections were mounted with DePex mounting medium (Gurr, BDH, Poole, Dorset, UK).

### Statistical analysis

Physiological data and qPCR results are reported as the mean ± the standard error (SE) for six mice. The differences between the mean values were tested for statistical significance by the two-tailed Student's *t *test.

## Results

### Dietary fat-induced obesity and insulin resistance in C57BL/6J mice

**Figure 1:**
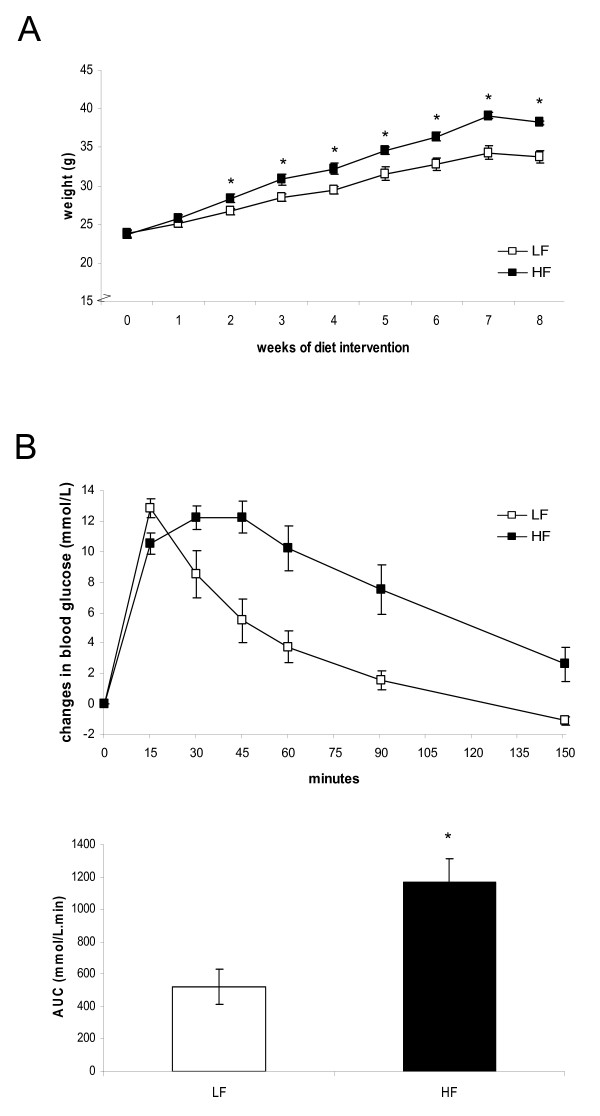
**Body weight and oral glucose tolerance test**. (A) Body weight gain of C57BL/6J mice during a low-fat or high-fat diet intervention of 8 weeks. (B) An oral glucose tolerance test was performed after 7 weeks of diet intervention. After an oral gavage of 100 mg glucose, blood glucose levels were monitored for 150 minutes. The changes in blood glucose levels (upper figure) and the area under he curve were calculated (lower figure). In (A) and (B), data are means ± SE. * p < 0.05. LF = low-fat diet, HF = high-fat diet.

To evaluate the effect of a Western-style humanized high-fat diet on the development of obesity and insulin resistance, C57BL/6J mice were fed a high-fat versus low-fat diet for eight weeks. Figure 1A shows that already after two weeks on the high-fat diet, C57BL/6J mice demonstrated a significantly higher weight gain than mice on the low-fat diet. Moreover, an oral glucose tolerance test performed after seven weeks of diet intervention showed that the high-fat diet induced a significantly higher glucose intolerance (Figure 1B), indicating development of insulin resistance. To determine whether these dietary effects are confounded by higher energy intake on the high-fat diet, we calculated food intake from the measurements of non-absorbable chromic oxide in feces (see Methods). We found that energy intake on the high-fat diet (10.8 ± 0.9 kcal/day) was even slightly lower, but not significantly different from that on the low-fat diet (11.9 ± 0.9 kcal/day). Thus, we conclude that not the amount, but solely the composition of the high-fat diet induced obesity and insulin resistance in C57BL/6J mice.

### Dietary fat-induced changes in small intestinal gene expression

**Figure 2:**
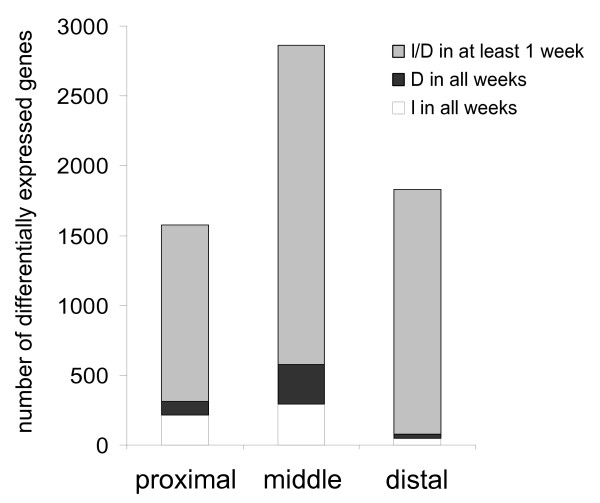
**Dietary fat-induced differential gene expression along the longitudinal axis of the small intestine**. For the proximal, middle and distal part of the small intestine, the numbers of genes that were differentially expressed in at least one week of diet intervention are plotted (grey bars). Among those are genes that were consistently up- (I) or down-regulated (D) on a high-fat diet (white and black bars, respectively).

After 2, 4 and 8 weeks of diet intervention, molecular changes between the low- and high-fat diet were analyzed for each part of the small intestine. The numbers of genes that were differentially expressed in at least one week of diet intervention are visualized in Figure 2. The highest numbers of genes, transiently (grey bars) as well as continuously differentially expressed during diet intervention (black and white bars), were found in the middle part of the small intestine. The most pronounced (-3 > fold change > +3) and consistent molecular changes throughout diet intervention are listed in Additional file 4. As microarray analyses in this study were performed on mouse samples that were pooled per diet group per time point, qPCR was used to verify differential gene expression of randomly selected genes in individual mouse samples. In all parts of the small intestine, but especially in the middle part, qPCR results were highly in accordance with the microarray data (see Additional file 5). In the proximal and distal part of the small intestine, variance between individual mouse samples was slightly higher and therefore the qPCR results were not always significant.

### Biological processes influenced in the small intestine by feeding a high-fat diet

**Figure 3:**
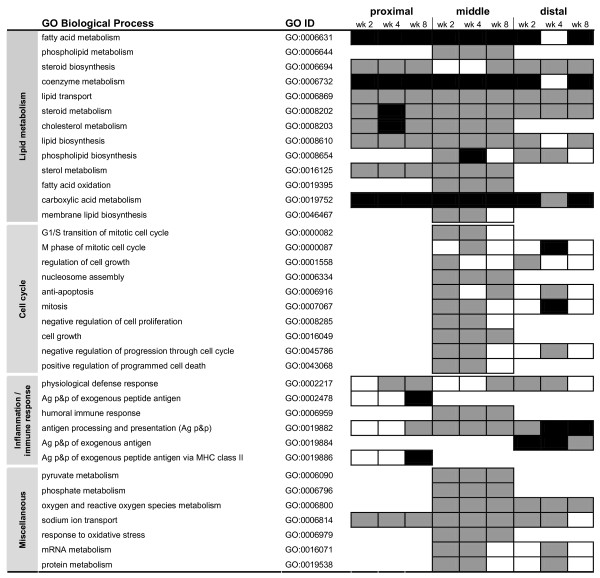
**Overrepresentation of GO Biological Process subsets in the small intestine during high-fat diet intervention**. In the ORA analysis all genes showing differential expression in at least one week of diet intervention were included. For the proximal, middle and distal part of the small intestine, GO Biological Process subsets with a FDR < 0.01 and a RawScore = 10 in at least one week of diet intervention are considered significantly overrepresented. Black boxes indicate 1.0E-31 < FDR < 1.0E-08; dark grey boxes indicate 1.0E-08 < FDR < 0.01; white boxes indicate FDR > 0.01, so not significant. An empty row indicates that this part of small intestine did meet the above mentioned selection criteria (i.e. FDR > 0.01 at all time points).

**Figure 4:**
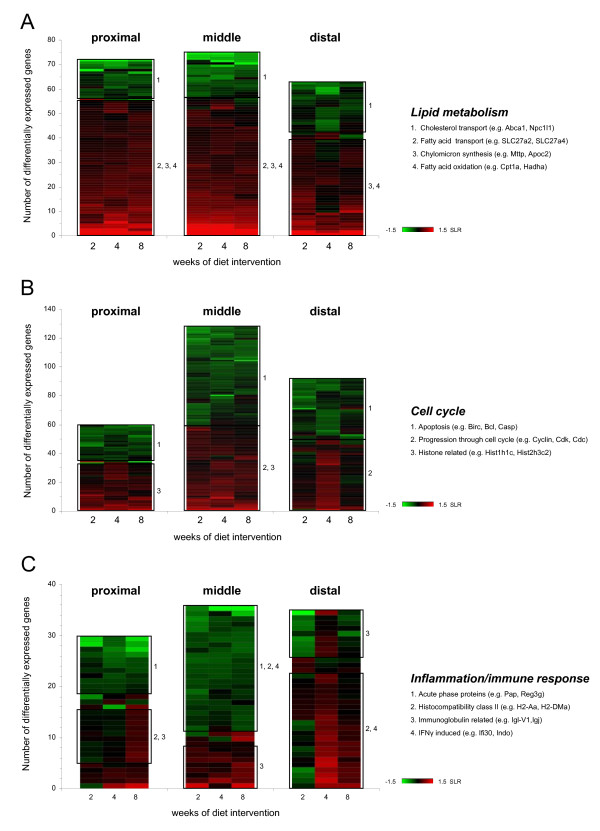
**Heat map diagrams of differentially expressed genes on a high-fat diet**. SLR of differentially expressed genes related to lipid metabolism (A), cell cycle (B) and inflammation/immune response (C) are clustered in a heat map diagram for the proximal, middle and distal part of the small intestine. Green and red indicate down- and up-regulation of gene expression, respectively. In general, three expression patterns can be distinguished; consistently up-regulated, consistently down-regulated or alternate up- and down-regulation during diet intervention. Amongst other genes that display similar expression patterns on a high-fat diet, the boxes include differentially expressed genes that share association with particular biological processes (numbered). Differentially expressed genes with a -0.3 > SLR > 0.3 in at least one week of diet intervention were included and the color scheme ranges from SLR -1.5 to 1.5.

We performed an overrepresentation analysis (ORA) for each part of the small intestine, to determine which biological processes were influenced by exposure of the small intestine to a high-fat diet. In the ORA analysis, all genes showing differential expression in at least one week of diet intervention were included (Figure 3). Additionally, heat map diagrams were created to display the number of differentially expressed genes that were annotated to the GO Biological Process terms listed in Figure 3 and to visualize the magnitude and direction of their differential expression (indicated by the SLR) (Figure 4).

ORA analysis showed that biological processes related to lipid metabolism were highly regulated by dietary fat in all parts of the small intestine. Additionally, the heat map diagrams in Figure 4A show that the up- or down-regulation of many lipid metabolism-related genes was very consistent in time and that dietary fat had the strongest effect in the proximal and middle part of the small intestine. In these parts, genes involved in fatty acid transport, chylomicron synthesis and especially fatty acid oxidation were highly up-regulated, whereas genes involved in cholesterol transport were down-regulated by dietary fat. In the distal part of the small intestine, similar regulation of fatty acid oxidation and cholesterol transport was seen, but less prominent than in the more proximal parts of the small intestine. These data indicate that processing of dietary fat is mainly accomplished in the duodenum and jejunum. However, the ileum is still capable of handling the overflow of fat.

**Figure 5:**
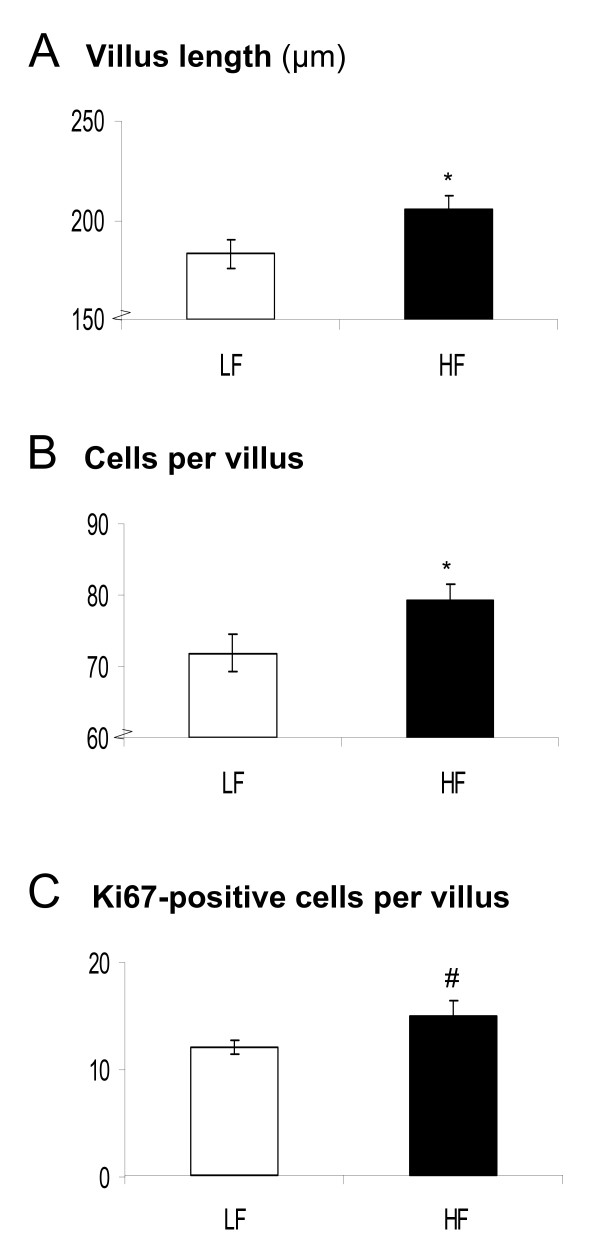
**Dietary fat-induced cell proliferation in the small intestine, analyzed by immunohistochemistry**. Immunohistochemistry was performed on distal small intestinal sections of C57BL/6J mice fed a low-fat or high-fat diet using Ki67-specific antibodies. Besides the villus length (A) and total number of villus cells (B), also the number of Ki67-positive cells per villus (C) were determined. Per mouse, 15 villi were counted and the mean values were calculated. * p < 0.05, # p = 0.07. LF = low-fat diet, HF = high-fat diet.

Furthermore, ORA analysis showed that dietary fat affected the regulation of cell cycle-related processes. This effect was most pronounced in the middle and distal parts of the small intestine. Cell proliferation seemed to be enhanced by dietary fat early in diet intervention, as at these time points genes that are essential for progression through cell cycle were up-regulated and genes involved in apoptosis were down-regulated (Figure 4B). Remarkably, after 8 weeks of diet intervention, hardly any differential gene expression related to cell cycle can be detected. To ensure that the dietary fat-induced modulations in cell cycle-related processes reflected proliferation of enterocytes and not immune cells (see below), we performed immunohistochemistry on the distal part of the small intestine. We found that villus length and the total number of cells per villus were significantly higher in mice fed the high-fat diet (Figure 5A and 5B). Additionally, we determined proliferation rates in the villi by specific staining of proliferation marker Ki67 (Figure 5C). Differences in Ki67-positive cells between the low-fat and high-fat diet were most pronounced at the base of the villi, showing a tendency towards an increased proliferation rate on a high-fat diet (p = 0.07). Together these data indicate that inhibition of apoptosis and increase in cell proliferation induced by dietary fat results in enlargement of small intestinal villi. This might be functional to extent the capacity of fat absorption.

GO Biological Process subsets related to inflammation/immune response were also overrepresented in small intestine after feeding a high-fat diet. Figure 4C showed that for many of these related genes the differential expression was not very consistent in time, especially in the proximal and distal part of the small intestine. However, in the middle part, a substantial number of genes showed a sustained down-regulation throughout high-fat diet intervention. Remarkably, many of these down-regulated genes are known to be interferon gamma (IFNγ)-inducible genes [19,20]. This continuous down-regulation of genes in the middle part of the small intestine suggests a diminished inflammatory response to the high-fat diet. However, it remains difficult to draw a definitive conclusion on inflammatory status of the overall small intestine, as hardly any gene showed a continuous up- or down-regulation in all parts of the small intestine.

### Differential gene expression of nuclear receptors and their target genes in the small intestine after feeding a high-fat diet

**Figure 6:**
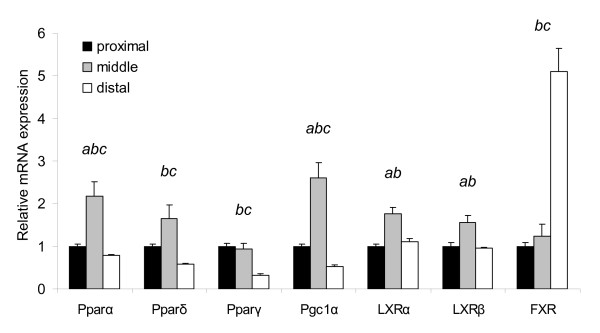
**Relative mRNA expression of nuclear receptors along the longitudinal axis of the small intestine under basal conditions**. For the proximal, middle and distal part of the small intestine, gene expression levels of Ppars, Lxrs and Fxr were determined after 2 weeks of low-fat diet intervention. qPCR data of the nuclear receptors are visualized as the mean expression of individual mice ± SE. Expression in the middle and distal part of the small intestine is relative to the expression in the proximal part, which was set to 1. *a*, *b*, and *c *indicate significant (p < 0.05, two-tailed Student's *t *test) differential gene expression between the distinct parts of the small intestine (*a *between the proximal and middle part, *b *between the middle and distal part and *c *between the proximal and distal part).

**Figure 7:**
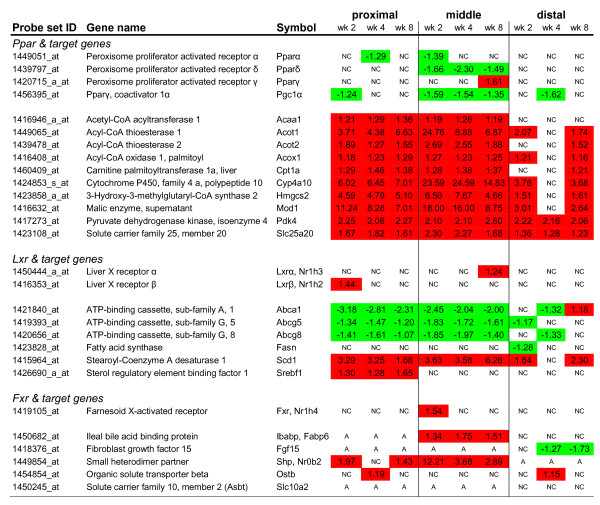
**Dietary fat-induced differential expression of nuclear (hormone) receptors and their target genes in the small intestine**. For the proximal, middle and distal part of the small intestine, differential gene expression (indicated by fold changes) of nuclear (hormone) receptors and their target genes was determined by microarray analysis, after 2, 4, and 8 weeks of high-fat diet intervention. Red and green boxes indicate a significant up- and down-regulation, respectively. NC = no change, A = absent.

Nutritional modulation of metabolic pathways is frequently mediated by nuclear receptors that act as ligand-activated transcription factors [21]. In the small intestine, we found that gene expression related to lipid metabolism-related processes was highly affected after exposure to a high-fat diet. As peroxisome proliferator activated receptors (Ppars) and liver X receptors (Lxrs) are commonly linked to these pathways, we selected these nuclear receptors to study their role in transcriptional regulation during high-fat diet intervention. Another metabolic nuclear receptor that might be a key regulator of the response to dietary fat is the farnesoid X-activated receptor (Fxr). This receptor is activated by bile acids [22], which are supposed to be elevated in the small intestinal lumen after feeding a high-fat diet to facilitate digestion. First, we determined the expression of the nuclear receptors along the longitudinal axis of the small intestine under basal conditions (on a low-fat diet) (Figure 6). For most nuclear receptors, the highest expression could be detected in the middle part of the small intestine where fat absorption is expected to be most pronounced. The highest *Fxr *expression was found in the ileum, the predominant site for absorption of bile acids. Except for the continuously down-regulation of *Pparδ *and co-activator *Pgc1α *in the middle part of the small intestine, the effect of a high-fat diet on nuclear receptor expression itself was minimal (Figure 7). However, even more informative for their regulatory involvement in lipid metabolism-related processes, is the ligand-specific activation pattern of the nuclear receptors. Therefore, we analyzed the differential expression of the target genes of the nuclear receptors, which reflects their activation by specific nutrients (primary diet effects; e.g. fatty acids) and/or diet-related metabolites (secondary diet effects; e.g. biliary cholesterol and bile acids). Pparα, -δ and -γ can all be activated by fatty acids and in general there seems to be a substantial overlap between the target genes of these nuclear receptors. In the small intestine, the activation of Pparα is most extensively studied and therefore we mainly focused on the differential expression of Pparα target genes as identified in Pparα null mice [23] (Figure 7). Our microarray data clearly show that Pparα is considerably activated on the high-fat diet, as numerous target genes were up-regulated especially in the proximal and middle part of the small intestine. As many of the target genes were also annotated to overrepresented lipid metabolism-related processes (Figure 3 and Figure 4A), we conclude that Pparα plays an important regulatory role in absorption and processing of the dietary fat in the small intestine.

Nuclear receptors Lxrα (Nr1h2) and Lxrβ (Nr1h3) are known to be activated by endogenous oxysterol metabolites. They control gene expression related to cholesterol and fatty acid metabolism. Surprisingly, for these biological processes we found quite different expression patterns of the target genes. Cholesterol transporters *ATP-binding cassette, subfamily A1, -G5 and -G8 *(*Abca1*, *Abcg5 *and *Abcg8*, respectively) were down-regulated by a high-fat diet, whereas *stearoyl-Coenzyme A desaturase 1 *(*Scd1*) and *sterol regulatory element binding factor *(*Srebf1*) were up-regulated. *Fatty acid synthase *(*Fasn*) showed hardly any regulation. These data suggest that on a high-fat diet the expression of these target genes is not exclusively controlled by these Lxr receptors. Other transcription factors and co-regulators might also be involved.

Based on the expression patterns of Fxr (Nr1h4), we expected the most pronounced target gene regulation in the distal part of the small intestine. However, in this part we only found a late down-regulation of *fibroblast growth factor 15 *(*Fgf15*), a signaling molecule that represses bile acid synthesis in the liver. Remarkably, the most pronounced differential gene expression of Fxr target genes was found in the middle part of the small intestine, namely a continuous up-regulation of *ileal bile acid binding protein *(*Ibabp*) and *small heterodimer partner *(*Shp*). These results suggest that on a high-fat diet the passive absorption of unconjugated bile acids increases in the middle part of the small intestine, while active absorption of bile acids in the distal part of the small intestine is probably decreased. Taken together, our data showed that Ppars, Lxrs and Fxr are substantially activated by the primary and secondary metabolites of a high-fat diet and might therefore play an important role in regulation of small intestinal lipid metabolism-related processes during high-fat diet intervention.

### Secretome analysis of differentially expressed genes in the small intestine after feeding a high-fat diet

**Figure 8:**
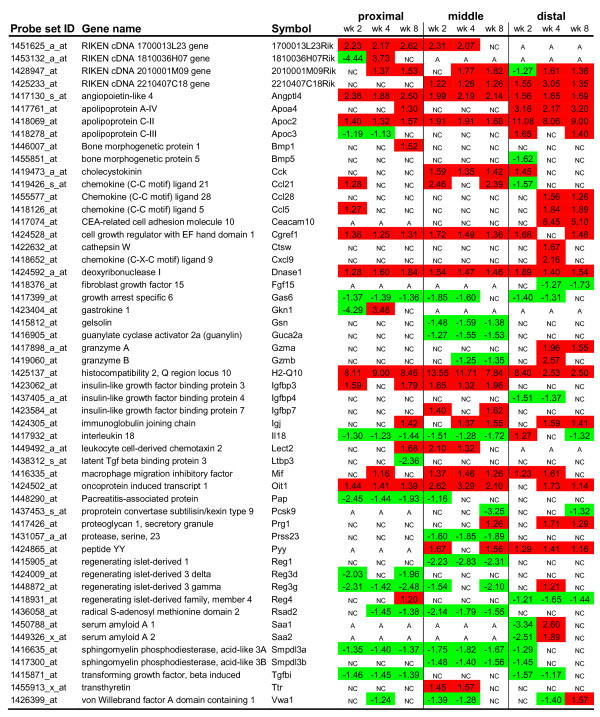
**Secretome analysis of differentially expressed genes in the small intestine during high-fat diet intervention**. Secretome analysis was performed for the proximal, middle and distal part of the small intestine and included differentially expressed genes with fold changes < -1.5 and > +1.5 in at least one week of diet intervention. Red and green boxes indicate a significant up- and down-regulation, respectively. NC = no change, A = absent.

Next to molecular changes that provoke local metabolic effects in the small intestine, we also studied the differential gene expression of (novel) small intestinal secreted proteins that are able to induce systemic effects. To identify the secreted proteins that are differentially expressed during high-fat diet intervention, we performed a secretome analysis. For this analysis, genes that were differentially expressed in at least one week of diet intervention (fold change >1.5) were additionally selected for their corresponding GO Cellular Component term 'extracellular region/space' (GO:0005576/GO:0005615) (Figure 8). Some of these selected genes showed a consistent differential gene expression throughout the small intestine and at all time points (e.g. *Angptl4*, *ApoC2*, *Dnase1*, *Cgref1*, *Gas6*, *H2-Q10*). Differential expression of others genes was restricted to a more specific part of the gut (e.g. *Cck*, *Gsn, Pap, Reg1*, *Fgf15*, *Ccl28*, *Pyy*). For several genes, also a time effect could be detected in their differential expression, as they showed early (e.g. *Igfbp4*, *Ttr*) or late phase (e.g. *Ccl5*, *Ccl28*, *Igj*, *Fgf15*) responses. Consistent with the ORA analysis data described above, many of the secreted proteins are related to lipid metabolism, especially chylomicron synthesis (e.g. ApoA4, ApoC2, ApoC3), and inflammation/immune response (e.g. several chemokines, H2-Q10, Il18, Mif, Rsad2, Saa1/2). Although we do not propose that all of these secreted proteins act as signaling molecules that provoke a systemic effect on peripheral organs, we consider genes related to inflammation/immune response and the gut hormones (e.g. Cck, Pyy) as promising candidates. Also genes with a pronounced differential gene expression, of which function is not completely elucidated yet (e.g. Angptl4, H2-Q10, Oit1, Smpdl3a/b) are potential interesting signaling molecules that might contribute to development of obesity and/or insulin resistance.

## Discussion

In this study, we demonstrated that C57BL/6J mice develop obesity and glucose intolerance on a high-fat diet that mimics the fatty acid composition of a Western-style human diet. It might seem surprising that despite the isoenergetic intake on the low- and high-fat diet, only mice on a high-fat diet become obese. However, this can (at least partly) be explained by the increased energy dissipation on the low-fat diet due to ATP requirement for the conversion of glucose to fatty acids. This process occurs in liver and adipose tissue of rodents [24], but is thought to be of minor importance in humans on a Western-style diet [25].

Microarray analyses of small intestinal mucosa showed that dietary fat had the most pronounced effect in the middle part of the small intestine. Furthermore, we found that many dietary fat-induced molecular changes are related to lipid metabolism, cell cycle and inflammation/immune response. Although it seems obvious that lipid metabolism with respect to fatty acid absorption and transport is changed in the small intestine after feeding a high-fat diet, it remains intriguing that also fatty acid oxidation is elevated. So far, there is no solid explanation for this, but we speculate that small intestinal fatty acid oxidation is enhanced to prevent toxic accumulation of free fatty acids in the enterocyte. In liver, muscle and adipose tissue, chronic inflammation is also considered to be a key player in the development of metabolic disorders [26]. In the small intestine, however, we found no conclusive evidence for an increased inflammatory status that might contribute to development of dietary fat-induced obesity and insulin resistance.

Further analysis of our microarray data revealed that nuclear receptors, such as Ppars, Lxr and Fxr play an important role in the regulation of small intestinal metabolism after feeding a high-fat diet. Besides this, we also found differential gene expression of signaling molecules that can be secreted from the small intestinal mucosa and provoke systemic effects. To determine the association of these dietary-fat induced changes in the small intestine with development of obesity and/or insulin resistance, we linked them to previous data that were mainly obtained by using null- and transgenic mice models (Table 1). We consider the molecular changes that point into the right direction (Table 1; shown in bold) to be potential small intestinal effectors in the etiology of the metabolic syndrome. This will now be further discussed. Recently, Tobin et al. reviewed the role of Ppars, Lxrs and Fxr in diseases that are related to the metabolic syndrome [27]. However, these transcription factors were mainly studied in liver, muscle and white adipose tissue and little is known so far about their small intestinal function. In our study, we found an elevated expression of many target genes of Ppars, especially of Pparα, which are involved in lipid metabolism-related processes, such as fatty acid transport and fatty acid oxidation. Previous studies showed that Pparα null mice become more obese on a high-fat diet than wild-type mice, but are resistant to dietary fat-induced insulin resistance [28,29]. With regard to our study this might suggest that an elevated activation of Pparα in the small intestine can contribute to development of insulin resistance on a high-fat diet. As in null mice, the lack of Pparα is associated with obesity it is hard to directly link our Pparα-related gene expression data to this metabolic disorder. However, Kondo et al. recently showed that a suboptimal activation of Pparα and therefore an impaired fatty acid oxidation in the small intestine of C57BL/6J mice might also contribute to the development of dietary fat-induced obesity [5]. Interestingly, the decreased expression of *Pgc1α *that we found in our study might point in the direction of a suboptimal activation of Pparα, as it is known that Pgc1α can cooperatively induce the expression of Pparα target genes and increase cellular fatty acid oxidation rates [30]. Moreover, a decreased expression of Pgc1α was previously already linked to an inefficient fatty acid oxidation [31]. The continuous down-regulation of *Pparδ *in the middle part of the small intestine might also be related to development of insulin resistance, as Pparδ null mice become more glucose intolerant on a high-fat diet [32]. Taken together, Ppars and their co-activator Pgc1α might be substantial small intestinal contributors to development of obesity and/or insulin resistance. For Lxr target genes *Abca1*, *Scd1 *and *Srebf1 *(encodes for sterol regulatory element binding protein 1c (Srebp1c)), a similar discrepancy as was seen for their small intestinal gene expression on a high-fat diet was reflected in the susceptibility of the corresponding null mice to develop obesity and/or insulin resistance (Table 1). Abca1 null mice become more glucose intolerant, whereas Scd1 and Srebp1c null mice are protected against these diet-induced metabolic disorders [33-35]. Therefore, dietary fat-induced down-regulation of *Abca1 *and up-regulation of *Scd1 *and *Srebf1 *might contribute to development of obesity and/or insulin resistance. The strong up-regulation of Fxr target gene *Shp *in the middle part of the small intestine might also be involved in development of these metabolic diseases, as previous null mice studies showed that mice lacking Shp are resistant to dietary fat-induced obesity and insulin resistance [36]. Moreover, the study of Wang et al. [36] implied that high expression levels of Shp might be correlated with decreased levels of Pgc1α. Our study also shows an inverse relationship between the expression of *Shp *and *Pgc1α*. Exploration of the small intestinal function of Abca1, Scd1, Srebp1c and Shp might further elucidate their potential role in development of obesity and/or insulin resistance.

**Table 1:** Obesity- and/or insulin resistance-associated genes and their expression in the small intestine of C57BL/6J mice fed a high-fat diet.

**Gene name (symbol)**	**Previous association with obesity and/or insulin resistance (ref.)**		**Transcriptional regulation in small intestine on a HF diet**
*Nuclear receptors & target genes*			
ATP-binding cassette, sub-family A, 1 (Abca1)	-/- mice; IR ▲	[45]	**↓ P, M, D**
Farnesoid X-activated receptor (Fxr, Nr1h4)	-/- mice; IR ▲	[46, 47]	minimal regulation, but target genes: ↑ P, M; ↓ **D**
Liver X receptor (LXRα/β, Nr1h2/3)	-/- mice; obesity & IR ▼	[48]	minimal regulation, but target genes: cholesterol metabolism ↓ P, M, D **fatty acid metabolism ↑ P, M**
Peroxisome proliferator activated receptor α (Pparα)	-/- mice; obesity ▲, IR ▼	[28, 29]	minimal regulation, but target genes: **↑ P, M, D**
Peroxisome proliferator activated receptor δ (Pparδ)	-/- mice; IR ▲	[32]	**↓, M**
Peroxisome proliferator activated receptor γ (Pparγ)	-/+ mice; IR ▼ Agonists (TZDs) used as anti-diabetic agents	[49, 50]	minimal regulation
Pparγ, coactivator 1α (Pgc1α)	-/- mice; obesity ▲, IR ▼	[51]	**↓ M**
Stearoyl-Coenzyme A desaturase 1 (Scd1)	-/- mice; obesity & IR ▼	[33, 34]	**↑ P, M, D**
Sterol regulatory element binding factor 1 (Srebf1)	-/- mice; obesity ▼	[35]	**↑ P**
Small heterodimer partner (Shp, Nr0b2)	-/- mice; obesity & IR ▼	[36]	**↑ P, M**

*Secreted (signaling) proteins*			
Angiopoietin-like 4 (Angptl4)	Tg mice; obesity ▼, IR ▲ Suppression by gut microbiota promotes triglyceride storage	[6]	**↑ P, M, D**
Fibroblast growth factor 15 (Fgf15)	Injection of Fgf15; glucose tolerance ▲	[41]	**↓ D**
Glucagon-like peptide-1 (Glp1)	High plasma levels; IR ▼	[12, 13]	↑ (slightly) M
Gastric inhibitory polypeptide (Gip)	High plasma levels; IR ▼	[13]	↑ (slightly) D
Insulin-like growth factor binding protein 3 (Igfbp3)	Tg mice; IR ▲	[42]	**↑ P, M**
Interleukin 18 (Il18)	-/- mice; obesity & IR ▲	[37]	**↓ P, M**
Macrophage migration inhibitory factor (Mif)	High plasma levels; obesity & IR ▲	[39]	**↑ M, D**

-/- mice: null mice, +/- mice: heterozygous mice, Tg mice: transgenic mice, IR: insulin resistance, P, M, D: proximal, middle, distal part of the small intestine, ▲: prone to, ▼: resistant to, ↑: up-regulated expression, ↓: down-regulated expression. In bold: dietary-fat induced molecular changes in the small intestine that point in the same direction as data from previous null- and transgenic mouse studies regarding development of metabolic syndrome.

Various signaling molecules that we found differentially expressed in the small intestine after exposure to a high-fat diet were previously associated with excessive weight gain and an impaired glucose homeostasis. A recent study showed that mice lacking the inflammatory cytokine interleukin 18 (Il18) have markedly increased body weight and are insulin resistant [37,38]. It is even suggested that Il18 possesses a glucose-lowering potential. The continuous decreased expression of Il18 in the proximal and middle part of the small intestine that we found after a high-fat diet intervention might suggest a role in dietary fat-induced obesity and insulin resistance. Next to Il18, macrophage inhibitory factor (Mif) is a pro-inflammatory cytokine that we found up-regulated in the small intestine by dietary fat. This increased expression might contribute to elevated plasma levels of Mif that are previously related to obesity and insulin resistance [39,40]. Furthermore, Fgf15 expression is thought to be inversely associated with development of insulin resistance [41], whereas a positive correlation is reported for expression of insulin-like growth factor binding protein 3 (Igfbp3) [42]. In accordance with these previous studies, we found a down-regulation of *Fgf15 *in the distal small intestine and an up-regulation of *Igfbp3 *in the proximal parts of small intestine after feeding a high-fat diet. Previous studies showed that suppression of Angptl4 by gut microbiota, was related to dietary fat-induced obesity [6]. In contrast, we found a continuous up-regulation of this gene in all parts of the small intestine. This implies that in our C57BL/6J mice, the gut microbiota was not able to suppress Angptl4 on a high-fat diet. Nevertheless, the mice still became obese. Therefore, we conclude that small intestinal Angptl4 is probably not a main contributor to development of obesity in our mouse model. Interestingly, however, studies of Mandard et al. showed that elevated levels of Angptl4 in blood are related to glucose intolerance [43]. This might indicate that the dietary-fat induced up-regulation of Angptl4 in the small intestine can provoke a systemic effect on development of insulin resistance. Moreover, a recent study of Desai et al. indicates that Angptl4 plays an important role in the small intestine [44]. They showed that Angptl4 null mice display severe intestinal pathology, especially abnormalities of intestinal lymphatics, which even deteriorated after feeding a high-fat diet. Further research will be required to more accurately elucidate the function of Angptl4, but also of Il18, Fgf15, Mif and Igfbp3 in the small intestine and their possible role in the etiology of the metabolic syndrome. For the incretin hormones, gastric inhibitory polypeptide and glucagon-like peptide-1, that are frequently associated with insulin sensitivity we found hardly any changes in gene expression on a high-fat diet. Therefore, in our study the incretins probably do not contribute to the development of dietary fat-induced insulin resistance in the C57BL/6J mice.

## Conclusion

During development of obesity and insulin resistance, biological processes related to lipid metabolism, cell cycle and inflammation/immune response are most strongly modulated in the small intestine of C57BL/6J mice fed a high-fat diet that mimics the fatty acid composition of a Western-style human diet. Our data further indicate that Ppars, Lxrs and Fxr are important metabolic regulators in the response of the small intestine to dietary fat. Next to these local small intestinal effects of a high-fat diet, we also found modulated expression of secreted proteins, such as Il18, Fgf15, Mif, Igfbp3 and Angptl4. These signaling molecules might provoke metabolic effects in liver, muscle and adipose tissue that underlie the development of the metabolic syndrome. Finally, as many of the dietary-fat induced molecular changes in the small intestine point in the same direction as data from previous null- and transgenic mouse studies, we propose these changes to be plausible effectors illustrating the role of the small intestine in the etiology of the metabolic syndrome.

## Competing interests

The authors declare that they have no competing interests.

## Authors' contributions

NW, MM and RM participated in the design and supervision of the study. HBV performed most experimental work, such as sample preparation and qPCR. MG and JJ hybridized the microarrays. PG and GH assessed the quality control of the microarrays and provided support for microarray analysis. NW performed microarray analysis and drafted, together with RM, the manuscript. GH and MM provided valuable feedback on the initial draft. All authors read and approved the final manuscript.

## Pre-publication history

The pre-publication history for this paper can be accessed here:



## Supplementary Material

Additional file 1 Diet composition. Composition of the low-fat and high-fat diets that were used in the diet intervention study in C57BL/6J mice. Click here for file

Additional file 2 Outline of the diet intervention study in C57BL/6J mice. After a 3-weeks acclimatization period on the low-fat purified (LF) diet, C57BL/6J mice were fed a LF or high-fat (HF) purified diet for 2, 4 and 8 weeks (n = 6 per diet group, per time point). Body weight was recorded weekly. After 7 weeks of diet intervention, an oral glucose tolerance test (OGTT) was performed on six mice per diet group. 'A' indicates that for both diets at week 2, 4 and 8, microarray analysis was performed on the proximal, middle and distal part of the small intestine (SI), using pooled RNA samples for each diet group. Click here for file

Additional file 3 Primer sequences. Overview of primer sequences that were used for qPCR verification of dietary fat-induced differential gene expression in the small intestine of C57BL/6J mice. Click here for file

Additional file 4 Genes showing a consistent differential expression in the small intestine of C57BL/6J mice in all weeks of diet intervention. List of genes with the most pronounced (fold changes < -3.0 and > +3.0 in at least one week of diet intervention) and consistent differential gene expression in the small intestine of C57BL/6J mice during diet high-fat intervention. Click here for file

Additional file 5 Verification of microarray results in individual mice by qPCR analysis. For the proximal (A), middle (B) and distal part of the small intestine (C), five genes that were found to be differentially expressed by microarray analysis were randomly selected and their expression was validated in individual mouse samples by qPCR. The qPCR data are visualized as the mean expression of all individual mice per diet group per time point ± SE, relative to the expression on the LF diet at week 2, which was set to 1. Only the results of the 18S normalization are shown as they are similar to the results obtained for the cyclophilin A normalization. White and black bars represent gene expression on the low- and high-fat diet, respectively. # significant differential gene expression indicated by MAS 5.0. * p < 0.05 (two-tailed Student's *t *test). Click here for file
