# Supplementary material for: The role of the small intestine in the development of dietary fat-induced obesity and insulin resistance in C57BL/6J mice
Source: BMC Med Genomics. 2008 May 6;1:14. doi: 10.1186/1755-8794-1-14 (PMC2396659; doi:10.1186/1755-8794-1-14)
Supplement: Additional file 1 — Diet composition. Composition of the low-fat and high-fat diets that were used in the diet intervention study in C57BL/6J mice. [file 1755-8794-1-14-S1.doc]

**Supplementary table S1. Diet composition.**

|  | **Low-fat (LF) diet** | | **High-fat (HF) diet** | |
| --- | --- | --- | --- | --- |
| Based on formula # | D12450B* | | D12451* | |
|  |  | |  | |
|  | **gm%** | ***kcal%*** | **gm%** | ***kcal%*** |
| Protein | 19 | *20* | 24 | *20* |
| Carbohydrate | 67 | *70* | 41 | *35* |
| Fat | 4 | *10* | 24 | *45* |
|  |  |  |  |  |
|  |  |  |  |  |
| **Ingredients** | **gm** | ***kcal*** | **gm** | ***kcal*** |
| Casein, lactic | 200 | *800* | 200 | *800* |
| L-Cystine | 3 | *12* | 3 | *12* |
|  |  |  |  |  |
| Corn Starch | 427.2 | *1709* | 72.8 | *291* |
| Maltodextrin | 100 | *400* | 100 | *400* |
| Sucrose | 172.8 | *691* | 172.8 | *691* |
|  |  |  |  |  |
| Cellulose, BW200 | 50 | *0* | 50 | *0* |
|  |  |  |  |  |
| Soybean Oil | 25 | *225* | 25 | *225* |
| Palm oil | 20 | *180* | 177.5 | *1598* |
|  |  |  |  |  |
| Mineral Mix S10026 | 10 | *0* | 10 | *0* |
| DiCalcium Phosphate | 13 | *0* | 13 | *0* |
| Calcium Carbonate | 5.5 | *0* | 5.5 | *0* |
| Potassium Citrate, 1 H2O | 16.5 | *0* | 16.5 | *0* |
|  |  |  |  |  |
| Vitamin Mix V10001 | 10 | *40* | 10 | *40* |
| Choline Bitartrate | 2 | *0* | 2 | *0* |
| **Total** | 1055 | *4057* | 858.15 | *4057* |
|  |  |  |  |  |

* Research Diets, Inc. (New Brunswick, NJ, USA)
