# Supplementary figures and images for: The role of the small intestine in the development of dietary fat-induced obesity and insulin resistance in C57BL/6J mice
Source: BMC Med Genomics. 2008 May 6;1:14. doi: 10.1186/1755-8794-1-14 (PMC2396659; doi:10.1186/1755-8794-1-14)

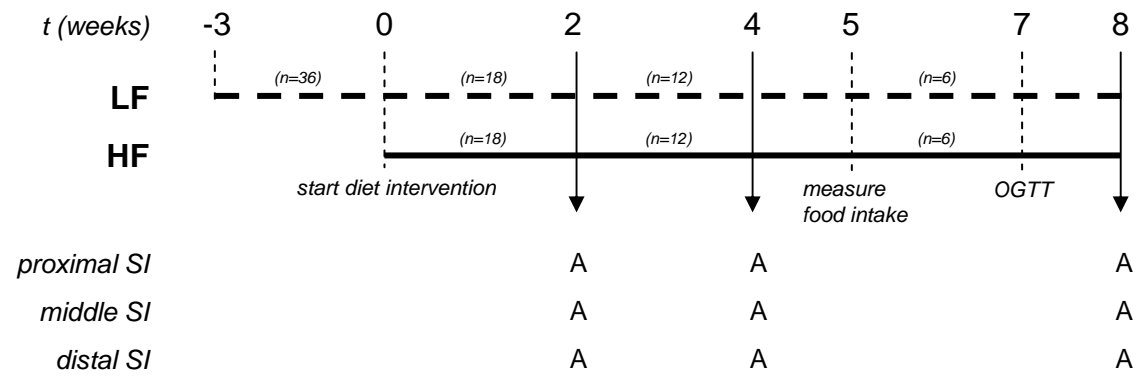

Supplement: Additional file 2 — Outline of the diet intervention study in C57BL/6J mice. After a 3-weeks acclimatization period on the low-fat purified (LF) diet, C57BL/6J mice were fed a LF or high-fat (HF) purified diet for 2, 4 and 8 weeks (n = 6 per diet group, per time point). Body weight was recorded weekly. After 7 weeks of diet intervention, an oral glucose tolerance test (OGTT) was performed on six mice per diet group. 'A' indicates that for both diets at week 2, 4 and 8, microarray analysis was performed on the proximal, middle and distal part of the small intestine (SI), using pooled RNA samples for each diet group. [file 1755-8794-1-14-S2.pdf]

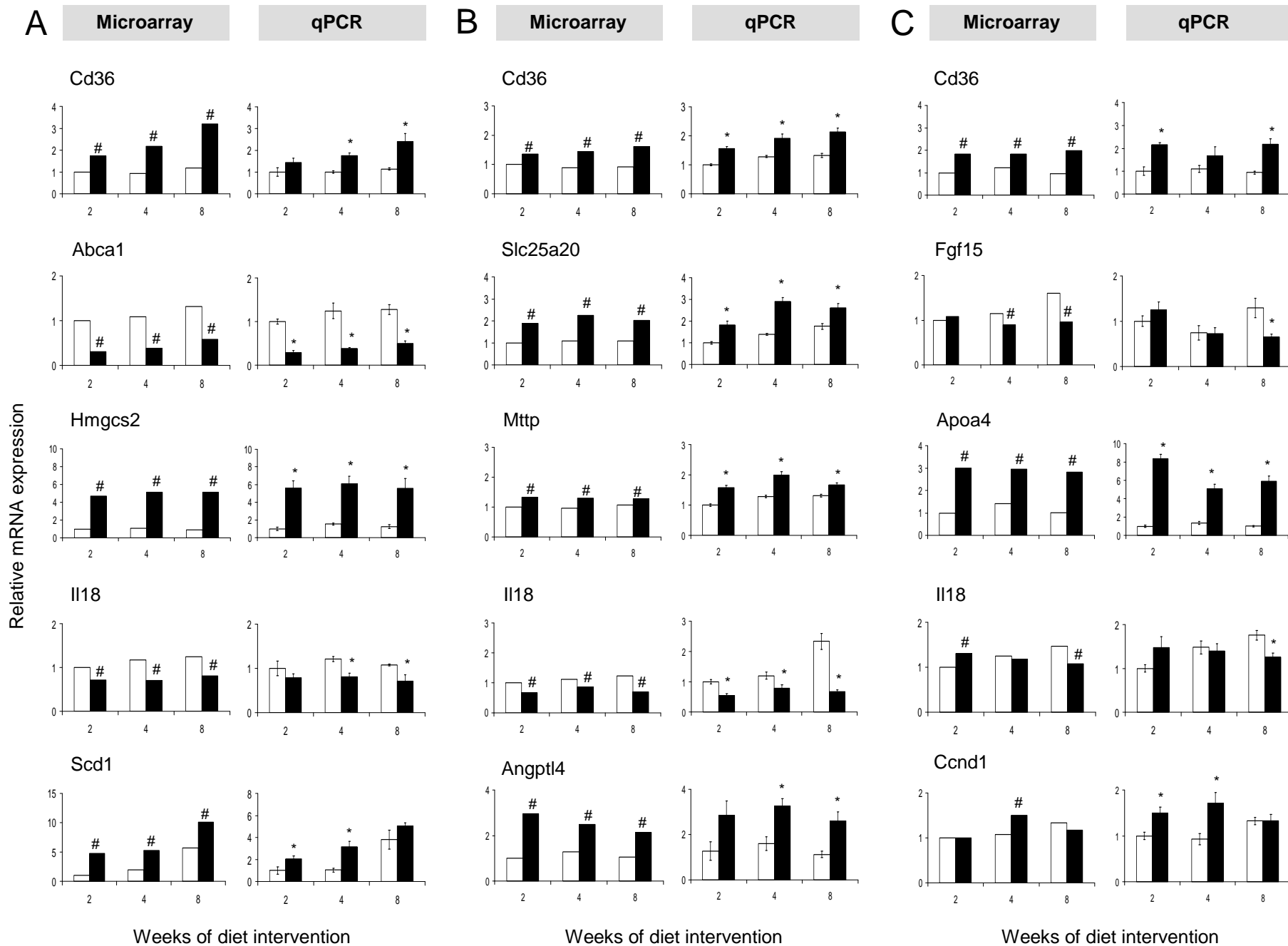

Supplement: Additional file 5 — Verification of microarray results in individual mice by qPCR analysis. For the proximal (A), middle (B) and distal part of the small intestine (C), five genes that were found to be differentially expressed by microarray analysis were randomly selected and their expression was validated in individual mouse samples by qPCR. The qPCR data are visualized as the mean expression of all individual mice per diet group per time point ± SE, relative to the expression on the LF diet at week 2, which was set to 1. Only the results of the 18S normalization are shown as they are similar to the results obtained for the cyclophilin A normalization. White and black bars represent gene expression on the low- and high-fat diet, respectively. # significant differential gene expression indicated by MAS 5.0. * p < 0.05 (two-tailed Student's t test). [file 1755-8794-1-14-S5.pdf]
