# Supplementary material for: The role of the small intestine in the development of dietary fat-induced obesity and insulin resistance in C57BL/6J mice
Source: BMC Med Genomics. 2008 May 6;1:14. doi: 10.1186/1755-8794-1-14 (PMC2396659; doi:10.1186/1755-8794-1-14)
Supplement: Additional file 3 — Primer sequences. Overview of primer sequences that were used for qPCR verification of dietary fat-induced differential gene expression in the small intestine of C57BL/6J mice. [file 1755-8794-1-14-S3.doc]

**Supplementary table S2. Primer sequences.**

| **Gene symbol** | **Forward primer** | **Reverse primer** |
| --- | --- | --- |
|  |  |  |
| Abca1 | 5’-CCCAGAGCAAAAAGCGACTC-3’ | 5’-ACCATCCATGCCTACAACAAAAGG-3’ |
| Angptl4 | 5’-CACCAATGTTTCCCCCAAT-3’ | 5’-CACGGAGGTCATGGTCTTG-3’ |
| Apoa4 | 5’-CAACAGGCTGAAGGCTACGAT-3’ | 5’-CGATTTTTGCGGAGACCTTGG-3’ |
| Ccnd1 | 5’-CAGAAGTGCGAAGAGGAGGTC-3’ | 5’-TCATCTTAGAGGCCACGAACAT-3’ |
| Cd36 | 5’-tccagccaatgcctttgc-3’ | 5’-tggagattacttttcagtgcagaa-3’ |
| Fgf15 | 5’-GCTCTGAAGACGATTGCCATC-3’ | 5’-TTCCTCCCTGAAGGTACAGTC-3’ |
| Fxr | 5’-GACCTCCACAACCAAGTTTTGC-3’ | 5’-TGATTTCCTGAGGCATTCTCTGTT-3’ |
| Hmgcs2 | 5’-TGGTGGATGGGAAGCTGTCTA-3’ | 5’-TTCTTGCGGTAGGCTGCATAG-3’ |
| Il18 | 5’-GACTCTTGCGTCAACTTCAAGG-3’ | 5’-CAGGCTGTCTTTTGTCAACGA-3’ |
| Lxrα | 5’-GCTCTGCTCATTGCCATCAG-3’ | 5’-TGTTGCAGCCTCTCTACTTGGA-3’ |
| Lxrβ | 5’-CGTGGTCATCTTAGAGCCAGA-3’ | 5’-GCTGAGCACGTTGTAGTGGAA-3’ |
| Mttp | 5’-ATACAAGCTCACGTACTCCACT-3’ | 5’-TCCACAGTAACACAACGTCCA-3’ |
| Pgc1α | 5’-AGACGGATTGCCCTCATTTGA-3’ | 5’-TGTAGCTGAGCTGAGTGTTGG-3’ |
| Pparα | 5’-TATTCGGCTGAAGCTGGTGTAC-3’ | 5’-CTGGCATTTGTTCCGGTTCT-3’ |
| Pparδ | 5’-TTGAGCCCAAGTTCGAGTTTG-3’ | 5’-CGGTCTCCACACAGAATGATG-3’ |
| Pparγ | 5’-CACAATGCCATCAGGTTTGG-3’ | 5’-GCTGGTCGATATCACTGGAGATC-3’ |
| Scd1 | 5’-CCGGAGACCCTTAGATCGA-3’ | 5’-TAGCCTGTAAAAGATTTCTGCAAACC-3’ |
| Slc25a20 | 5’-CCGAAACCCATCAGTCCGTTTAA-3’ | 5’-ACATAGGTGGCTGTCCAGACAA-3’ |
|  |  |  |
