# Supplementary material for: The role of the small intestine in the development of dietary fat-induced obesity and insulin resistance in C57BL/6J mice
Source: BMC Med Genomics. 2008 May 6;1:14. doi: 10.1186/1755-8794-1-14 (PMC2396659; doi:10.1186/1755-8794-1-14)
Supplement: Additional file 4 — Genes showing a consistent differential expression in the small intestine of C57BL/6J mice in all weeks of diet intervention. List of genes with the most pronounced (fold changes < -3.0 and > +3.0 in at least one week of diet intervention) and consistent differential gene expression in the small intestine of C57BL/6J mice during diet high-fat intervention. [file 1755-8794-1-14-S4.doc]

**Supplementary table 3.**  **Genes showing a consistent differential expression in the small intestine of C57Bl/6J mice in all weeks of diet intervention.**

|  |  |  | **Fold change** | | |
| --- | --- | --- | --- | --- | --- |
| **Probe set ID** | **Gene name** | **Symbol** | wk 2 | wk 4 | wk 8 |
|  |  |  |  |  |  |
| ***proximal*** |  |  |  |  |  |
| 1416632_at | malic enzyme, supernatant | Mod1 | 11.24 | 8.28 | 7.01 |
| 1425137_a_at | histocompatibility 2, Q region locus 10 | H2-Q10 | 8.11 | 9.00 | 8.46 |
| 1436169_at | RIKEN cDNA C730029A08 gene | C730029A08Rik | 7.26 | 3.27 | 3.56 |
| 1424853_s_at | cytochrome P450, family 4, subfamily a, polypeptide 10 | Cyp4a10 | 6.02 | 6.45 | 7.01 |
| 1448700_at | G0/G1 switch gene 2 | G0s2 | 5.43 | 5.35 | 9.25 |
| 1423858_a_at | 3-hydroxy-3-methylglutaryl-Coenzyme A synthase 2 | Hmgcs2 | 4.59 | 4.79 | 5.10 |
| 1449065_at | acyl-CoA thioesterase 1 | Acot1 | 3.71 | 4.38 | 6.63 |
| 1419622_at | UDP glucuronosyltransferase 2 family, polypeptide B5 | Ugt2b5 | 3.41 | 2.87 | 3.66 |
| 1415964_at | stearoyl-Coenzyme A desaturase 1 | Scd1 | 3.29 | 3.25 | 1.68 |
| 1418538_at | KDEL endoplasmic reticulum protein retention receptor 3 | Kdelr3 | 2.95 | 4.00 | 2.99 |
| 1424167_a_at | phosphomannomutase 1 | Pmm1 | 2.35 | 2.13 | 3.29 |
| 1433626_at | phospholipid scramblase 4 | Plscr4 | 2.31 | 2.75 | 3.76 |
| 1429286_at | RIKEN cDNA 1190003M12 gene | 1190003M12Rik | 1.42 | 1.79 | 5.82 |
| 1449907_at | beta-carotene 15,15'-monooxygenase | Bcmo1 | -9.71 | -14.52 | -13.36 |
| 1418787_at | mannose binding lectin (C) | Mbl2 | -5.50 | -16.68 | -8.11 |
| 1421840_at | ATP-binding cassette, sub-family A, member 1 | Abca1 | -3.18 | -2.81 | -2.31 |
| 1417651_at | cytochrome P450, family 2, subfamily c, polypeptide 29 | Cyp2c29 | -2.60 | -3.16 | -2.48 |
| 1435370_a_at | carboxylesterase 3 | Ces3 | -2.33 | -3.81 | -2.99 |
| ***middle*** |  |  |  |  |  |
| 1449065_at | acyl-CoA thioesterase 1 | Acot1 | 24.76 | 8.88 | 6.87 |
| 1424853_s_at | cytochrome P450, family 4, subfamily a, polypeptide 10 | Cyp4a10 | 23.59 | 24.59 | 14.83 |
| 1416632_at | malic enzyme, supernatant | Mod1 | 18.00 | 16.00 | 8.75 |
| 1425137_a_at | histocompatibility 2, Q region locus 10 | H2-Q10 | 13.55 | 11.71 | 7.84 |
| 1449854_at | nuclear receptor subfamily 0, group B, member 2 | Nr0b2 | 12.21 | 3.68 | 2.89 |
| 1424266_s_at | expressed sequence AU018778 | AU018778 | 6.77 | 6.54 | 4.59 |
| 1423858_a_at | 3-hydroxy-3-methylglutaryl-Coenzyme A synthase 2 | Hmgcs2 | 6.50 | 7.67 | 4.66 |
| 1448700_at | G0/G1 switch gene 2 | G0s2 | 6.28 | 7.62 | 8.94 |
| 1431688_at | hypothetical LOC73899 | LOC73899 | 5.94 | 2.01 | 4.56 |
| 1418538_at | KDEL endoplasmic reticulum protein retention receptor 3 | Kdelr3 | 5.58 | 4.59 | 4.20 |
| 1421040_a_at | glutathione S-transferase, alpha 2 (Yc2) | Gsta2 | 5.10 | 3.03 | 2.57 |
| 1419692_a_at | leukotriene C4 synthase | Ltc4s | 4.82 | 3.76 | 3.76 |
| 1419618_at | butyrobetaine (gamma), 2-oxoglutarate dioxygenase 1 | Bbox1 | 4.76 | 5.46 | 3.89 |
| 1427347_s_at | tubulin, beta 2 | Tubb2 | 4.69 | 4.17 | 2.69 |
| 1417812_a_at | laminin, beta 3 | Lamb3 | 4.66 | 4.06 | 3.53 |
| 1419622_at | UDP glucuronosyltransferase 2 family, polypeptide B5 | Ugt2b5 | 4.35 | 4.20 | 4.00 |
| 1456558_s_at | expressed sequence C87977 | C87977 | 4.35 | 2.91 | 2.71 |
| 1432790_at | RIKEN cDNA 9030218A15 gene | 9030218A15Rik | 4.17 | 2.10 | 1.65 |
| 1423436_at | glutathione S-transferase, alpha 3 | Gsta3 | 4.08 | 3.46 | 3.56 |
| 1417415_at | solute carrier family 6, member 3 | Slc6a3 | 3.68 | 5.86 | 5.17 |
| 1418848_at | aquaporin 7 | Aqp7 | 3.66 | 3.39 | 4.32 |
| 1415964_at | stearoyl-Coenzyme A desaturase 1 | Scd1 | 3.63 | 3.58 | 6.28 |
| 1430780_a_at | phosphomannomutase 1 | Pmm1 | 3.63 | 4.03 | 2.11 |
| 1452277_at | RIKEN cDNA 6330406P08 gene | 6330406P08Rik | 3.63 | 3.05 | 2.48 |
| 1429298_at | dimethylarginine dimethylaminohydrolase 1 | Ddah1 | 3.48 | 2.45 | 1.53 |
| 1459030_at | --- | --- | 3.39 | 3.51 | 3.39 |
| 1424962_at | transmembrane 4 superfamily member 4 | Tm4sf4 | 3.36 | 3.32 | 2.51 |
| 1420673_a_at | acyl-Coenzyme A oxidase 2, branched chain | Acox2 | 3.01 | 2.87 | 2.33 |
| 1426452_a_at | RAB30, member RAS oncogene family | Rsb30 | 3.01 | 2.91 | 2.01 |
| 1448777_at | minichromosome maintenance deficient 2 | Mcm2 | 2.71 | 3.05 | 2.89 |
| 1433626_at | phospholipid scramblase 4 | Plscr4 | 2.71 | 4.08 | 5.17 |
| 1424502_at | oncoprotein induced transcript 1 | Oit1 | 2.62 | 3.29 | 2.10 |
| 1459059_at | RIKEN cDNA 2010308F09 gene | 2010308F09Rik | 2.11 | 5.39 | 1.28 |
| 1449907_at | beta-carotene 15,15'-monooxygenase | Bcmo1 | -34.54 | -30.91 | -19.84 |
| 1418787_at | mannose binding lectin (C) | Mbl2 | -14.32 | -17.88 | -8.00 |
| 1424265_at | N-acetylneuraminate pyruvate lyase | Npl | -5.66 | -6.77 | -6.59 |
| 1416050_a_at | scavenger receptor class B, member 1 | Scarb1 | -4.82 | -4.17 | -3.39 |
| 1450167_at | RAB37, member of RAS oncogene family | Rab37 | -4.29 | -3.16 | -1.97 |
| 1450392_at | ATP-binding cassette, sub-family A, member 1 | Abca1 | -3.41 | -2.60 | -1.92 |
| 1434736_at | hepatic leukemia factor | Hlf | -3.27 | -3.14 | -2.62 |
| 1418382_at | adenomatosis polyposis coli down-regulated 1 | Apcdd1 | -2.95 | -3.25 | -2.22 |
| 1436021_at | RIKEN cDNA A930031D07 gene | A930031D07Rik | -2.79 | -2.45 | -3.51 |
| 1416432_at | 6-phosphofructo-2-kinase/fructose-2,6-biphosphatase 3 | Pfkfb3 | -2.41 | -3.05 | -3.27 |
| 1418979_at | RIKEN cDNA 9030611N15 gene | 9030611N15Rik | -2.36 | -2.35 | -2.17 |
| 1438610_a_at | Crystallin, zeta | Cryz | -2.36 | -4.41 | -1.58 |
| 1435370_a_at | carboxylesterase 3 | Ces3 | -2.11 | -3.01 | -2.46 |
| ***distal*** |  |  |  |  |  |
| 1418069_at | apolipoprotein C-II | Apoc2 | 11.08 | 8.06 | 9.00 |
| 1425137_a_at | histocompatibility 2, Q region locus 10 | H2-Q10 | 8.40 | 2.53 | 2.50 |
| 1422846_at | retinol binding protein 2 | Rbp2 | 3.43 | 2.28 | 3.10 |
| 1417761_at | apolipoprotein A-IV | Apoa4 | 3.16 | 2.17 | 3.20 |
| 1425233_at | RIKEN cDNA 2210407C18 gene | 2210407C18Rik | 1.55 | 3.05 | 1.35 |
| 1449907_at | beta-carotene 15,15'-monooxygenase | Bcmo1 | -3.63 | -3.39 | -2.30 |
| 1424265_at | N-acetylneuraminate pyruvate lyase | Npl | -2.38 | -3.46 | -2.17 |
| 1418174_at | D site albumin promoter binding protein | Dbp | -2.08 | -3.43 | -1.89 |
|  |  |  |  |  |  |

Fold changes are < -3.0 and > +3.0 in at least one week of diet intervention.
